# Construction and Analysis of Gene Co-Expression Networks in *Escherichia coli*

**DOI:** 10.3390/cells7030019

**Published:** 2018-03-08

**Authors:** Wei Liu, Li Li, Xuhe Long, Weixin You, Yuexian Zhong, Menglin Wang, Huan Tao, Shoukai Lin, Huaqin He

**Affiliations:** 1 School of Life Sciences, Fujian Agriculture and Forestry University, Fuzhou 350002, China; xhlong2015@sina.com (X.L.); ywx19971016@163.com (W.Y.); yuexianz@163.com (Y.Z.); 1170539009@fafu.edu.cn (M.W.); taohuan@fafu.edu.cn (H.T.); 2 Institute of Health Service and Medical Information, Academy of Military Medical Sciences, Beijing 100850, China; lili10010304@163.com; 3 Key Laboratory of Loquat Germplasm Innovation and Utilization, Putian University, Putian 351100, China

**Keywords:** *Escherichia coli*, gene co-expression network, annotation

## Abstract

Network-based systems biology has become an important method for analyzing high-throughput gene expression data and gene function mining. *Escherichia coli* (*E. coli*) has long been a popular model organism for basic biological research. In this paper, weighted gene co-expression network analysis (WGCNA) algorithm was applied to construct gene co-expression networks in *E. coli*. Thirty-one gene co-expression modules were detected from 1391 microarrays of *E. coli* data. Further characterization of these modules with the database for annotation, visualization, and integrated discovery (DAVID) tool showed that these modules are associated with several kinds of biological processes, such as carbohydrate catabolism, fatty acid metabolism, amino acid metabolism, transportation, translation, and ncRNA metabolism. Hub genes were also screened by intra-modular connectivity. Genes with unknown functions were annotated by guilt-by-association. Comparison with a previous prediction tool, EcoliNet, suggests that our dataset can expand gene predictions. In summary, 31 functional modules were identified in *E. coli*, 24 of which were functionally annotated. The analysis provides a resource for future gene discovery.

## 1. Introduction

*Escherichia coli* (*E. coli*) is an abundant bacteria in the intestine of humans and animals. It is a single-celled prokaryote that is widely used as a model organism in biology research. The *E. coli* genome size is about 4.64 M and encodes 5416 genes. Well-developed microarray technology has been used to investigate genome-wide gene expression thanks to its low cost. For convenience, we refer to gene as the corresponding probesets on the microarray throughout the manuscript when focusing on genes rather than probesets. There is extensive *E. coli* transcriptome data deposited in the public databases. These data include gene expression data under various conditions, such as different nutrients, growing stages, and gene mutations [1]. Scientists have been endeavoring to mine transcriptional regulation networks, gene expression networks, and protein–protein interaction networks by mathematical models [2]. It is still a challenge to reanalyze and discover the underlying information or knowledge within high-throughput data. Through the Google Scholar search engine, we found that the application of weighted gene co-expression network analysis (WGCNA) to *E. coli* has not yet been comprehensively reported. Although the metabolic network of *E. coli* has been constructed [3,4], only one recent study mentioned the construction of an *E. coli* gene network from 524 arrays, which focused on the statistical aspect by comparing four algorithms, including WGCNA [5]. Another study applied a community detection algorithm to the network of interactions identified with the context likelihood of relatedness (CLR) method from 730 arrays [6]. Three other studies have investigated the regulatory network of *E. coli* using hundreds of microarrays [7,8,9]. The EcoliNet database, which aims to provide function information for *E. coli* genes, collects co-functional gene networks for *E. coli* from seven distinct types of data, including co-expression pattern data from 132 microarrays [10].

The aims of our analysis were as follows: (1) to integrate the independent datasets, which will help to overcome the limitations caused by sample size and statistical method bias; (2) to summarize the thousands of genes on the array into tens of modules, which will help simplify data complexity and clarify the *E. coli* biological functions by modules, systematically; and (3) to provide organization or connection information within module genes, which may infer the potential function of unannotated genes by guilt-by-association rationale [11]. 

Here, the gene co-expression network for *E. coli* was constructed from 1391 microarrays. Thirty-one modules were identified, representing various aspects of *E. coli* biological functions. Hub genes bearing high connectivity may play roles in module function. Unknown genes were also annotated.

## 2. Results

### 2.1. A Gene Co-Expression Network for E. coli Was Successfully Constructed

**Figure 1:**
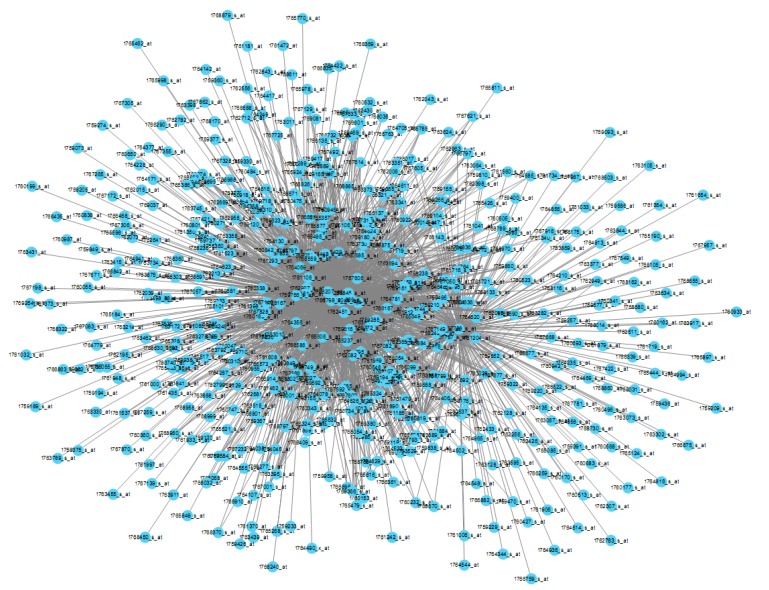
A representative network view of the constructed *Escherichia coli* (*E. coli*) gene co-expression network. Each node represents a probe and each line denotes the gene expression correlation between the two nodes.

The biggest gene co-expression network to date has been constructed (Figure 1). The top 2000 highly connected probe pairs were visualized by Cytoscape. The highest connected gene within the whole network is probe 1761661_s_at, which is annotated as an intergenic region; however, it localizes within the putative adhesin gene ycgV as inferred from its physical coordinate. Also, probe 1761661_s_at is highly connected in the Black module, whose members are enriched with plasma membrane protein-coding genes and the two-component system related genes (Table 1). 

**Figure 2:**
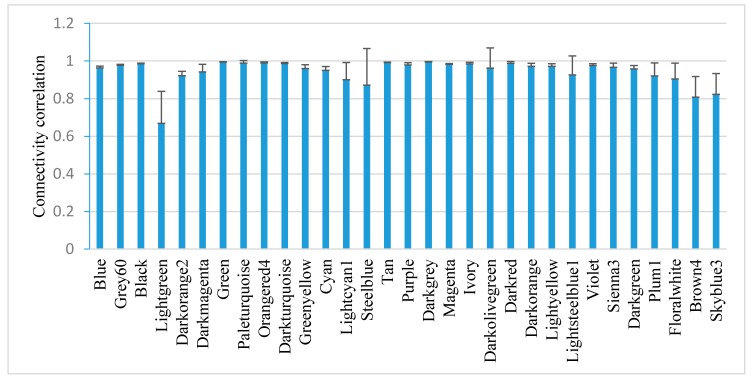
Correlation of intramodule connectivity for each module after sampling 1000 times (mean ± SD). Connectivity was calculated by randomly sampling half of the 1391 samples 1000 times.

WGCNA identified 31 stable co-expressed modules within which the genes have a similar expression pattern (Figure 2). To test the stability of the modules, the connectivity correlations between the original one and the one sampled 1000 times were calculated and expressed as the mean ± SD. All modules, except the Lightgreen module, show an average connectivity correlation smaller than 0.8. The most stable module is the Darkgrey and the least stable is the Steelblue.

### 2.2. Each Module Performs Distinct Functions

The database for annotation, visualization, and integrated discovery (DAVID) online tool was used to characterize the function of these identified modules. Twenty-four of them were annotated with significant Gene Ontology (GO) or Kyoto Encyclopedia of Genes and Genomes (KEGG) terms (Table 1). *E. coli* functions can be categorized into relatively independent functional modules. For instance, Blue is associated with secretion, whose coding proteins localize outside the cell. Magenta and Lightyellow both encode the peptidoglycan-based cell wall, but Lightyellow involves fatty acid oxidation and Magenta involves amine biosynthesis. Seven of these modules did not detect any function: Floralwhite, Lightgreen, Lightsteelblue1, Purple, Skyblue3, Steelblue, and Violet, corresponding to 34, 109, 37, 276, 45, 59, and 53 probesets, respectively. Among them, Floralwhite, Lightgreen, Lightsteelblue1, Skyblue3, and Steelblue are the eight least stable modules. The absence of DAVID terms being detected may be due to their relatively fewer probesets and lower module stability.

### 2.3. E. coli Hub Gene Screening

**Figure 3:**
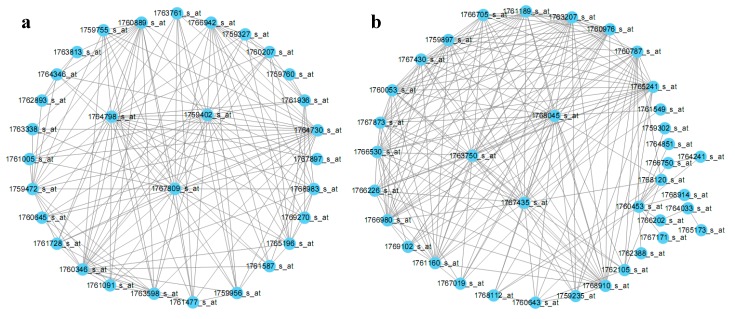
Module hub probes visualized in the modules Darkorange (**a**) and Darkred (**b**). The top 200 highly connected probe pairs for the two modules were visualized by Cytoscape.

In *E. coli* gene co-expression modules, each gene has a different contribution, that is, different connectivity. The higher the connectivity, the more important the gene in the module. Therefore, the contribution of a gene to a module can be simply described by its connectivity. The hub genes were screened by their connectivity in each module [12]. The screened hub genes are shown in Table 2. For instance, the top 200 connections of the Darkorange and Darkred modules are visualized in Figure 3.

### 2.4. Module-Based Analysis of Gene Expression Variation

**Figure 4:**
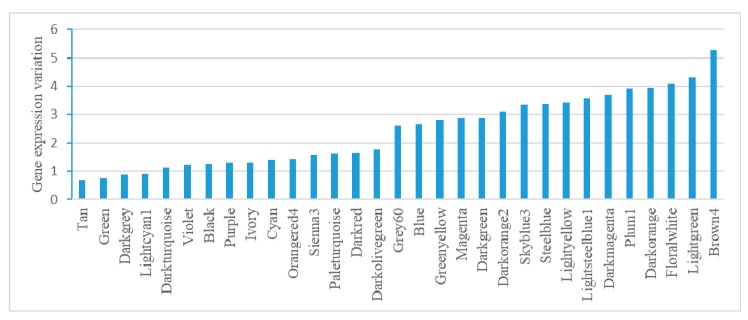
Gene expression variation analysis in *E. coli* modules. Variation was calculated as the average relative standard deviation for each gene in a module.

Analysis of gene expression variation in each module found modules performing housekeeping functions. Tan, Green, and Darkgrey have relatively low gene expression variation (lowest three), suggesting their housekeeping functions, such as ncRNA metabolism, translation, and lipopolysaccharide biosynthesis. Brown4, Darkorange, and Plum1 have relatively high gene expression variation (top three), mainly corresponding to stress responses, such as iron transmembrane transporter activity, pathogenesis, and anaerobic respiration (Figure 4).

### 2.5. Module-Based Analysis of Gene Connectivity

**Figure 5:**
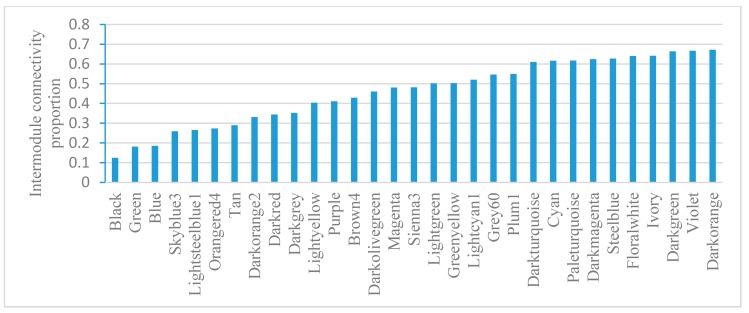
Intermodule connectivity proportion in *E. coli*. The intermodule connectivity/total connectivity value was calculated for every gene; then, the average value for each module was visualized.

Module gene connectivity includes intramodule connectivity and intermodule connectivity. Intramodule connectivity represents the relationship between genes within a specific module. Intermodule connectivity equals the total network connectivity minus intramodule connectivity, indicating the contribution of genes in coordinating functions across modules. Module-based gene connectivity analysis revealed that Black has the lowest intermodule connectivity while Darkorange has the highest intermodule connectivity (Figure 5). Darkorange is enriched with pathogenesis genes. Connectivity analysis indicates its important roles in coordinating module functions.

### 2.6. Modules That Correlate with Experimental Conditions

Modules can be independent units that perform biological functions. Linking the modular gene expression with experimental conditions may help to discover modules functioning in specific conditions. For example, inorganic phosphate limitation induces the highest Darkorange expression (Table S3). Darkorange is associated with pathogenesis and may be involved in responses to Pi availability and in the coordination of its virulence gene response [13]. Several modular genes are critical genes that respond to Pi limitation as reported, such as sepZ (1759760_s_at), escV (1761005_s_at), EspB (1765196_s_at), alkaline phosphatase (1767525_s_at), and Ler (1761795_s_at). Many other genes have been reported to be associated with virulence, such as intimin (1760207_s_at) [14], SepQ (1759472_s_at), SepL (1764730_s_at), EscC (1767809_s_at), EscD (1767897_s_at) (Table S4). 

The highest Darkred expression is induced when *E. coli* is cultured with bovine serum albumin (BSA) at the logarithmic growth phase [15]. Darkred is associated with flagella, which are important for biofilm formation by *E. coli*. Many Darkred genes are related to flagella, such as flgF (1759235_at), fliN (1765241_s_at), fliG (1766530_s_at), and flgG (1767435_s_at) (Table S4). Seventeen genes are annotated as hypothetical proteins. Our results may provide information for mining novel flagellum-related genes.

### 2.7. Comparison with Previous E. coli Networks

To compare our modules with those identified by a community detection algorithm [6], we overlapped the module functions and the Green and Darkred module member genes. Among the 13 modules from the Trevino study, 11 had GO annotations and all 11 modules were covered by our study with the same biological function (Table S5). We found 77 overlapping genes between the structural constituent of ribosome genes (the total was 106 in the Trevino study) and the Green module (Table S6). Another example is the 72 flagella genes, which were identified as the most significant GO-enriched community in the Trevino study. In our study, the Darkred module was annotated as flagellum and contains 87 potential genes that may be associated with flagella. Fifty-six genes overlap between these two lists (Table S7). In our study, the flagellum module also contains some genes annotated as hypothetical proteins. 

Some databases provide network-based *E. coli* gene function predictions, such as EcoliNet. An example here is the Darkred module, where all module genes should be associated with flagella. The 11 genes with unknown function were submitted to EcoliNet for function prediction, which found that 9 of these genes are predicted to be associated with flagella (Table S8). These results show that our analysis can expand the candidate gene list for flagella.

## 3. Discussion

WGCNA has been extensively applied for gene co-expression network construction in many species. The application in human brain transcriptome analysis revealed co-expressed modules in different brain regions [16]. In plant research, WGCNA identified cell-type specific and endoderm differentiation-associated gene co-expression modules [17]. Here, we analyzed 1391 *E. coli* microarrays and identified 24 modules with biological functional annotations. As *E. coli* is a simple organism, the results are easy to interpret. Traditional microarray analysis methods compare the control and treatment group, or multiple groups by ANOVA. When the data contains enormous amounts of samples, it is hard to analyze. WGCNA helps to reduce data dimension/complexity to only several modules that are complementary to the traditional analysis method. WGCNA does not require prior knowledge, so it may provide information for genome annotation.

*E. coli* biological functions can be categorized into four types: metabolism, ion transport, behavior, and pathogenesis. Metabolism modules include amino acid metabolism (Brown4, Darkturquoise, Magenta), carbohydrate metabolism (Black), lipopolysaccharide biosynthesis (Darkgreen, Darkgrey), sulfur metabolism (Darkolivegreen, Orangered4), and glycerol metabolism (Paleturquoise). Ion transport modules are Ivory and Grey60. The behavior module is Darkred. The pathogenesis modules are Blue, Darkmagenta, and Darkorange. Each module has a different function, suggesting the robustness of WGCNA.

Gene function prediction by co-expression approaches is based on the similarity of gene expression profiles across multiple samples or conditions. More data would help to increase the predictive power [18]. Therefore, all the data under various conditions were pooled to obtain more general module results. The advantages of our study is that it is the largest sample size to date. WGCNA assumes that groups of genes that encode proteins primarily or exclusively and function together in a pathway or molecular complex will be coordinately transcribed. The modules may help to annotate genes with unknown function. The Darkred module example shows that our results are comparable to those previously reported.

Further identification of hub genes may help to reveal their important role in modules. For instance, the Darkorange SepD hub gene may play roles in *E. coli* pathogenesis [19]; Black Z4148 is annotated as a hypothetical gene that might participate in carbohydrate metabolism, inferred from module function. Our results provide information for gene function and experimental validation. Lastly, the module-based analysis has revealed potential housekeeping and stress response modules by gene expression variation. Individual genes with high intermodule gene connectivity may suggest a role as module coordinator. For example, Darkorange may sense signals from the environment and play roles in intracellular signaling. In sum, our analysis is the first to construct an *E. coli* gene co-expression network and provides clues for candidate genes suitable for experimental validation.

## 4. Materials and Methods 

### 4.1. Data Preparation

A total of 165 GEO Series (GSE) datasets from *E. coli* were downloaded from the National Center for Biotechnology Information Gene Expression Omnibus (GEO) database [1]. These datasets include 1391 GSM files, representing 1391 biological samples. Details about these experiments and samples are provided in Tables S1 and S2. To improve the reproducibility and simplicity of the data analysis, only Affymetrix *E. coli* Genome 2.0 Array data were processed. Raw gene chip data were analyzed by Expression Console (Version 1.2) by MAS5.0 method. Probe-level gene expression data were retrieved.

### 4.2. Weighted Gene Co-Expression Network Analysis (WGCNA)

The WGCNA package was run on R (Version 2.3.2) to construct a gene co-expression network and identify modules with the following parameters: networkType = ‘signed’, softPower = 12, minModuleSize = 30, deepSplit = 4. Briefly, a weighted correlation network was created by calculating the correlation coefficients with the power β [20]. The β = 12 was chosen as a saturation level for a soft threshold of the correlation matrix based on the criterion of approximate scale-free topology. The weighted network was transformed into a network of topological overlap (TO), an advanced co-expression measurement that considers not only the correlation of two genes with each other but also the extent of their shared correlations across the weighted network [20]. The TO matrix was then used to group highly co-expressed genes by hierarchical clustering. Finally, a dynamic tree cut algorithm was used to cut the hierarchical clustering tree, and modules were defined as the branches resulting from this tree cutting [21]. Each module was summarized using singular value decomposition so that each module eigengene (ME) represents the first principal component of the module expression profiles [20]. Thus, ME explains the maximum amount of variation of the module expression levels and is considered the most representative gene expression in a module.

Module stability was tested as the average correlation between the original connectivity and the connectivity from 1391 samples that were randomly sampled 1000 times. The process was run for every module.

### 4.3. Module-Based Qualitative and Quantitative analysis

Genes in each of the identified co-expressed modules were annotated by the database for annotation, visualization and integrated discovery (DAVID 6.7) [22]. In DAVID, the term enrichment was defined as the Benjamini-adjusted Fisher exact test *p*-value. To visualize the whole network or specific module, the WGCNA exportNetworkToCytoscape function and Cytoscape tool [23] were used.

For gene expression variation analysis, the relative standard deviation for each gene in a module was calculated and the average values for each module were visualized. For connectivity analysis, the intermodule connectivity/total connectivity value was calculated for every gene and then the average value for each module was visualized.

### 4.4. Comparison of Gene Prediction Using Published Data

The *E. coli* network constructed by Trevino [6] and the EcoliNet gene-prediction tool [10] were used to compare candidate gene lists. For direct comparison, we overlapped the modules with the same biological function. Genes with unknown function in Darkred module were submitted to EcoliNet [10] for prediction.

## Figures and Tables

**Table 1:** Gene Ontology (GO) and Kyoto Encyclopedia of Genes and Genomes (KEGG) annotation of the 24 gene co-expression modules identified in *E. coli*.

Module (No. Probes)	GO Term (Benjamini-Adjusted *p*-Value)			KEGG (Adjusted *p*-Value)
	Biological Process	Cellular Component	Molecular Function	
Black (3608)	Carbohydrate catabolic process (8.5 × 10^−12^)	Plasma membrane (4.9 × 10^−30^)	Cation binding (6.8 × 10^−4^)	Two-component system (3.7 × 10^−31^)
Blue (1215)	Biological adhesion (3.6 × 10^−2^)	External encapsulation structure part (2.7 × 10^−2^)	—	Bacterial secretion system (3.4 × 10^−17^)
Brown4 (30)	Siderophore metabolic process (3.1 × 10^−5^)	—	Iron transmembrane transporter activity (6.5 × 10^−6^)	Lysine degradation (4.5 × 10^−4^)
Cyan (193)	Oxidation-reduction (9.3 × 10^−4^)	Anchored to membrane (2.9 × 10^−2^)	—	—
Darkgreen (154)	—	—	Purine nucleoside binding (1.9 × 10^−3^)	Lipopolysaccharide biosynthesis (1.8 × 10^−4^)
Darkgrey (78)	Lipopolysaccharide biosynthetic process (5.4 × 10^−23^)	Organelle inner membrane (8.3 × 10^−3^)	Cell surface antigen activity, host-interacting (8.5 × 10^−29^)	Lipopolysaccharide biosynthesis (1.2 × 10^−11^)
Darkmagenta (48)	—	Viral capsid (2.0 × 10^−5^)	—	—
Darkolivegreen (49)	Sulfate metabolic process (2.3 × 10^−33^)	—	Sulfate transmembrane transporter activity (2.1 × 10^−5^)	Sulfur metabolism (5.4 × 10^−12^)
Darkorange (74)	Pathogenesis (2.1 × 10^−5^)	—	—	Pathogenic *E. coli* infection (6.7 × 10^−9^)
Darkorange2 (999)	Phosphonate transport (2.5 × 10^−6^)	Peptidoglycan-based cell wall (5.9 × 10^−11^)	—	ABC transporters (3.8 × 10^−15^)
Darkred (87)	Behavior (2.1 × 10^−63^)	Flagellum (4.1 × 10^−51^)	Motor activity (3.3 × 10^−32^)	Flagellar assembly (1.4 × 10^−67^)
Darkturquoise (79)	Cellular amino acid biosynthetic process (1.4 × 10^−66^)	External encapsulating structure (1.2 × 10^−2^)	Acetolactate synthase activity (1.4 × 10^−6^)	Valine, leucine, and isoleucine biosynthesis (2.5 × 10^−11^)
Green (678)	Translation (2.6 × 10^−64^)	Ribosome (2.4 × 10^−59^)	Structural constituent of ribosome (5.8 × 10^−61^)	Ribosome (2.4 × 10^−56^)
Greenyellow (258)		peptidoglycan-based cell wall (2.1 × 10^−2^)		
Grey60 (101)	Iron ion transport (4.5 × 10^−2^)	—	Nucleoside binding (4.5 × 10^−3^)	ABC transporters (1.8 × 10^−2^)
Ivory (35)	Transition metal ion transport (1.4 × 10^−14^)	—	Iron ion binding (2.5 × 10^−10^)	Biosynthesis of siderophore group nonribosomal peptides (3.4 × 10^−10^)
Lightcyan1 (36)	Protein folding (4.2 × 10^−6^)		Zinc ion binding (9.2 × 10^−4^)	—
Lightyellow (93)	Fatty acid oxidation (1.7 × 10^−2^)	Peptidoglycan-based cell wall (2.3 × 10^−2^)	Acyl carrier activity (1.5 × 10^−10^)	Benzoate degradation via CoA ligation (3.0 × 10^−2^)
Magenta (483)	Amine biosynthetic process (9.9 × 10^−12^)	Peptidoglycan-based cell wall (2.4 × 10^−3^)	Nucleoside binding (2.0 × 10^−4^)	Glycine, serine and threonine metabolism (1.2 × 10^−4^)
Orangered4 (38)	Nitrogen compound biosynthetic process (2.8 × 10^−24^)	—	Anthranilate synthase activity (1.7 × 10^−2^)	Thiamine metabolism (4.4 × 10^−10^)
Paleturquoise (56)	Glycerol metabolic process (6.3 × 10^−7^)	—	Glycerol-3-phosphate dehydrogenase activity (8.0 × 10^−5^)	Glycerophospholipid metabolism (1.4 × 10^−3^)
Plum1 (40)	Anaerobic respiration (1.3 × 10^−2^)	Nitrate reductase complex (3.8 × 10^−2^)	—	—
Sienna3 (46)	Anaerobic respiration (1.1 × 10^−2^)	—	Metal cluster binding (2.2 × 10^−6^)	Butanoate metabolism (1.5 × 10^−5^)
Tan (295)	ncRNA metabolic process (4.3 × 10^−13^)	Plasma membrane (3.5 × 10^−6^)	RNA methyltransferase activity (7.1 × 10^−5^)	Mismatch repair (3.8 × 10^−5^)

— represents no significant GO or KEGG terms detected.

**Table 2:** Hub genes and their encoding proteins of the gene co-expression modules in *E. coli*.

Module	Gene	Encoding Protein
Black	Z4148	Hypothetical protein
Blue	ECs1057	Hypothetical protein
Brown4	fhuF	Ferric iron reductase protein
Cyan	ECs2033	Hypothetical protein
Darkgreen	elaA	Hypothetical protein
Darkgrey	rfbB	dTDP-glucose 4,6 dehydratase, NAD(P)-binding
Darkmagenta	c1590	Tail component of prophage
Darkolivegreen	ssuC	Alkanesulfonate transporter permease
Darkorange	ECs4574	SepD
Darkorange2	c4484	Aldolase
Darkred	ECs2660	Flagella biosynthesis protein FliZ
Darkturquoise	ECs5231	Ornithine carbamoyltransferase subunit I
Green	rpsN	30S ribosomal protein S14
Greenyellow	c0944	Hypothetical protein
Grey60	ECs1840	Hypothetical protein
Ivory	fecR	Anti-sigma transmembrane signal transducer for ferric citrate transport; periplasmic FecA-bound ferric citrate sensor and cytoplasmic FecI ECF sigma factor activator
Lightcyan1	ECs3950	RNA polymerase sigma factor RpoD
Lightyellow	pepN	Aminopeptidase
Magenta	usg	Semialdehyde dehydrogenase
Orangered4	ECs1854	OMP decarboxylase
Paleturquoise	yagF	CP4-6 prophage
Plum1	yebT	MCE domain protein
Sienna3	ECs2379	Hypothetical protein
Tan	ECs4128	Acetyl-CoA carboxylase biotin carboxylase subunit

## Supplementary Materials

Supplementary materials are available online http://www.mdpi.com/2073-4409/7/3/19/s1. Table S1: *E. coli* datasets used in our study; Table S2: 1391 *E. coli* samples’ characterization; Table S3: Samples that have the highest or lowest ME value in a specific module; Table S4: Probes modular assignment, connectivity, and their functional annotation; Table S5: Comparison of seven communities with GO annotations identified in the Trevino study at fmin = 4; Table S6: Comparison of ribosome modular genes identified by the Trevino study and our study; Table S7: Overlap of flagellum modular genes identified by the Trevino study and our study; Table S8: 11 genes with unknown function in Darkred module were submitted to EcoliNet for function prediction.
